# Supplementary material for: High Genetic Diversity in Third-Generation Cephalosporin-Resistant Escherichia coli in Wastewater Systems of Schleswig-Holstein
Source: Pathogens. 2024 Jan 20;13(1):90. doi: 10.3390/pathogens13010090 (PMC10820474; doi:10.3390/pathogens13010090)
Supplement: Supplementary file 1 [file pathogens-13-00090-s001.zip › Supplementary Figure 1.pdf]

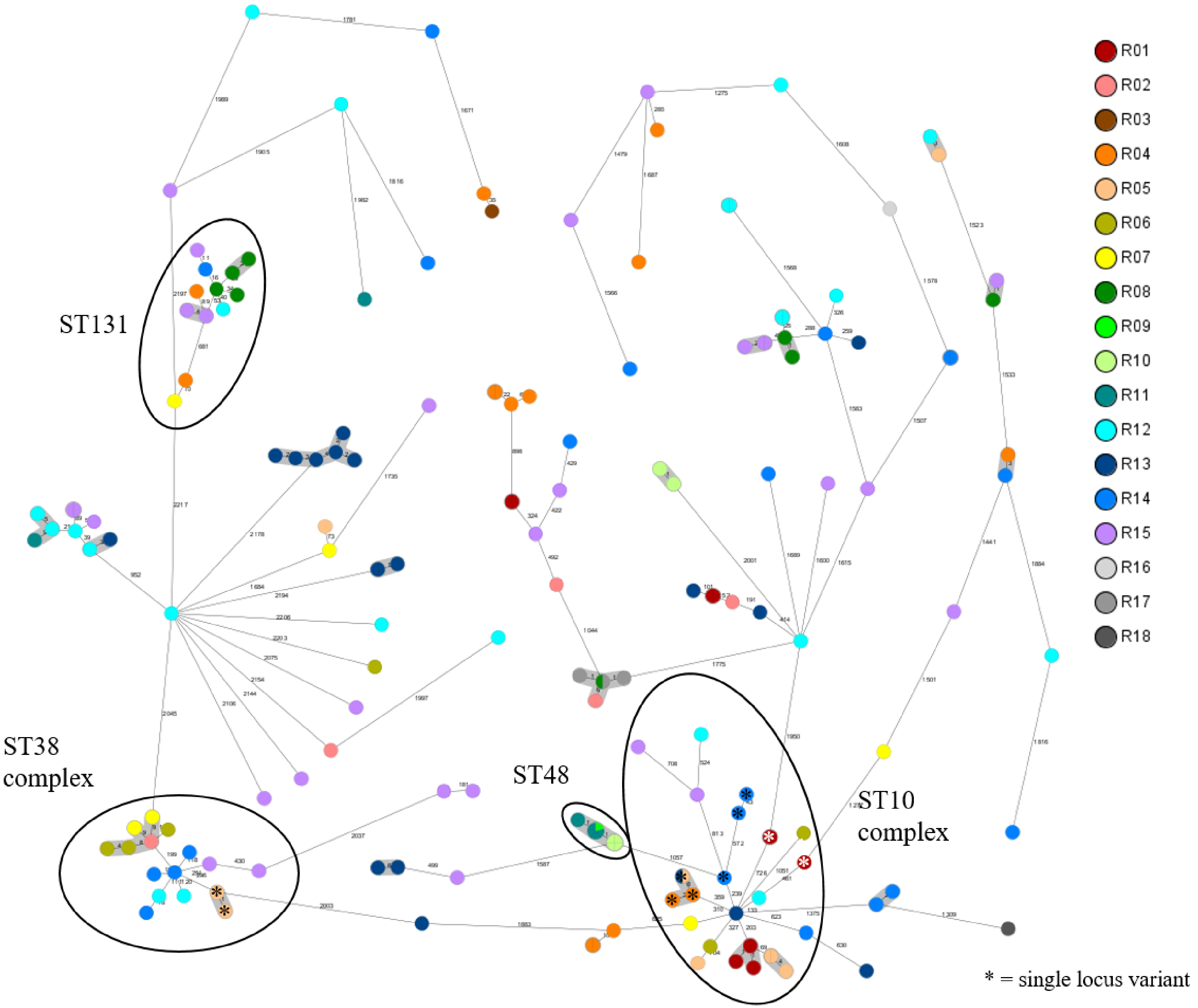

Supplementary Figure 1: clonal relations for all sequenced *E. coli* isolates from the four WWTPs. Isolates are colored according to their phenotypic resistance type (R01 – R18). The four most common sequence types (STs, n > 10) are circled and labeled accordingly. Isolates with a single locus variance to one of the STs are marked with stars.
