# Supplementary material for: High Genetic Diversity in Third-Generation Cephalosporin-Resistant Escherichia coli in Wastewater Systems of Schleswig-Holstein
Source: Pathogens. 2024 Jan 20;13(1):90. doi: 10.3390/pathogens13010090 (PMC10820474; doi:10.3390/pathogens13010090)
Supplement: Supplementary file 1 [file pathogens-13-00090-s001.zip › Supplementary Figure 2.pdf]

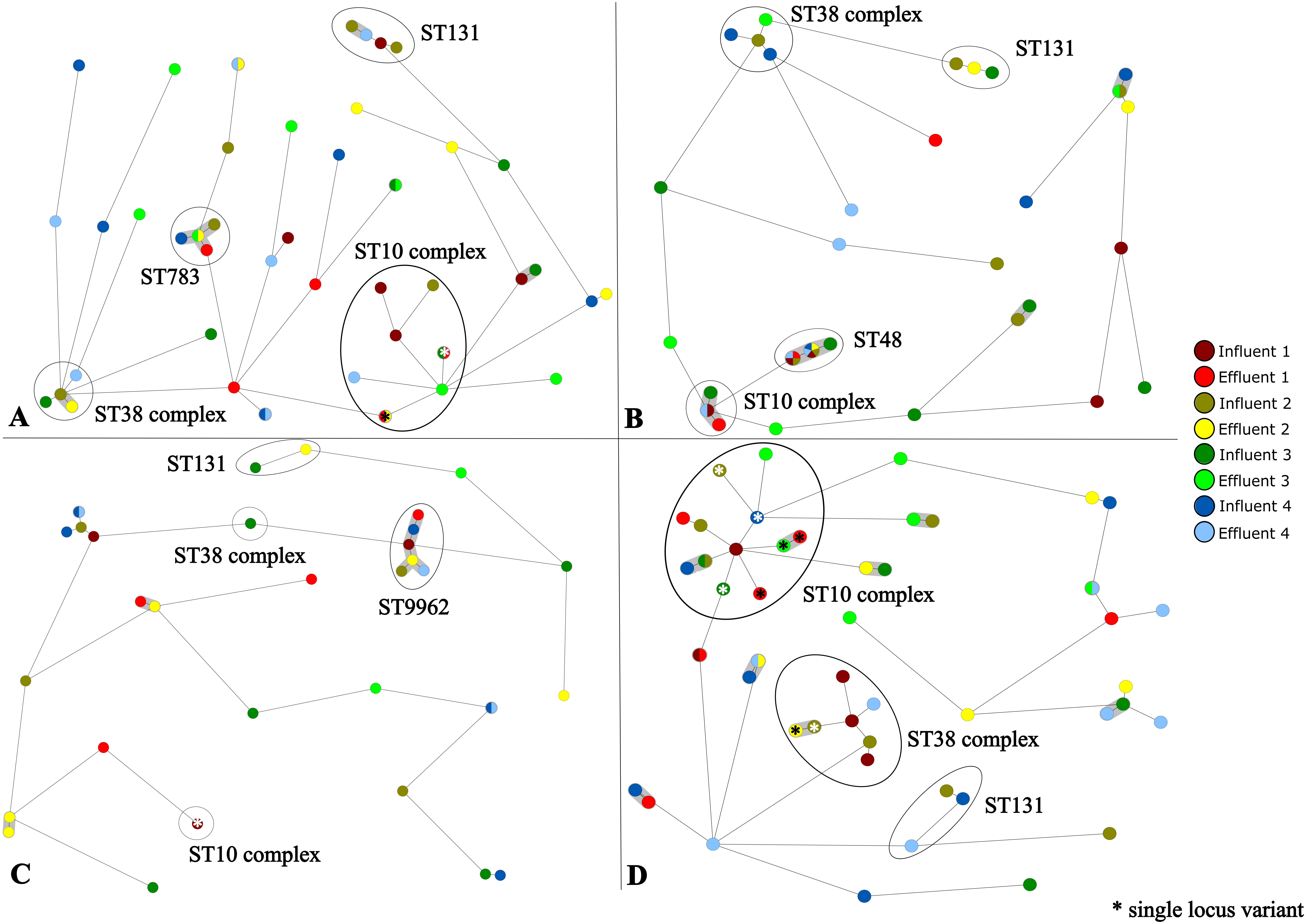

Supplementary Figure 2: clonal relations for all sequenced *E. coli* isolates from the WWTPs 1 (A), 2 (B), 3 (C) and 4 (D). Isolates are colored according to the sampling date and sampling site. Influent and effluent of consecutive days were taken at 24 h intervals. The four most common sequence types (STs; n>10) as well as WWTP specific STs are circled and labeled accordingly. Isolates with a single locus variance to one of these STs are marked with stars.
